# Facilitating Resident Interaction Through Robot-Delivered Trivia in Assisted Living Settings

**DOI:** 10.1093/geroni/igaf122.4312

**Published:** 2025-12-31

**Authors:** Shannon Power, Anne Adams, Lydia Burton, Kasey Smith, Jenay Beer

**Affiliations:** University of Georgia, Athens, Georgia, United States; SimpleC LLC, Marietta, Georgia, United States; University of Georgia, Athens, Georgia, United States; SimpleC LLC, Marietta, Georgia, United States; University of Georgia, Athens, Georgia, United States

## Abstract

With high dementia prevalence and few professional caregivers, socially assistive robots (SAR) offer a promising alternative to support social and cognitive engagement among older adults in residential care. This study included observation of SAR-facilitated trivia for older residents in assisted living settings. The SAR was a 4-ft humanoid Softbank Robotics Pepper robot with a chest-mounted tablet. Trivia interactions were designed to support cognitive engagement through curated content, timed sequences, and natural language paired with expressive animations. Each session included an introduction, trivia Q&A, discussion prompts, and closing. A total of 82 customized trivia items were created, with tailored speech (pitch, speed, emotion), 70+ matching gestures, expressions, jokes, laughter, and music. Seven observation sessions were conducted across two facilities. The naturalistic setting allowed residents (sample range: 2-16) and staff (sample range: 0-5) to join/leave freely. 289 observational segments were documented by six researchers via a standardized template. Segments were coded using the CREATE Model of Aging and Technology, across four non-exclusive categories: user, technology(robot), environment, and task(trivia). Observations revealed active engagement, prompting frequent interactions between Resident+Task (e.g., answer questions, freq=51), Resident+Resident (e.g., laughing, side conversation, freq=39), and Resident+Robot (e.g., asking robot questions, freq=28). Staff+Task interactions were least frequent (e.g., prompting, repeating questions, freq=16). Data suggest that the trivia tasks were engaging, the robot facilitated much of the social interaction, and the staff were able to monitor and supplement the interaction. Future design recommendations include AI-optimization of speech clarity, volume, and pacing to accommodate differences in resident cognitive abilities.

